# The 5^th ^anniversary of the "Universal Protocol": pitfalls and pearls revisited

**DOI:** 10.1186/1754-9493-3-14

**Published:** 2009-07-01

**Authors:** Philip F Stahel, Philip S Mehler, Ted J Clarke, Jeffrey Varnell

**Affiliations:** 1 Department of Orthopaedic Surgery, Denver Health Medical Center, University of Colorado Denver, School of Medicine, 777 Bannock Street, Denver, CO 80204, USA; 2 Department of Neurosurgery, Denver Health Medical Center, University of Colorado Denver, School of Medicine, 777 Bannock Street, Denver, CO 80204, USA; 3 Department of Patient Safety and Quality, Denver Health Medical Center, University of Colorado Denver, School of Medicine, 777 Bannock Street, Denver, Denver, CO 80204, USA; 4 Department of Internal Medicine, Denver Health Medical Center, University of Colorado Denver, School of Medicine, 777 Bannock Street, Denver, Denver, CO 80204, USA; 5 Colorado Physician Insurance Company (COPIC), Headquarters, Denver, CO 80230, USA

## The Universal Protocol

The publication date of this editorial marks the 5^th ^anniversary of the "Universal Protocol" which became a mandatory quality standard introduced by the *Joint Commission *on July 1, 2004 [1-3]. The Universal Protocol – designed to ensure correct patient identity, correct scheduled procedure, and correct surgical site – consists of the following three components:

1. A pre-procedure verification process.

2. Surgical site marking.

3. Surgical "time out" immediately prior to starting the procedure.

The pre-procedure verification process and surgical site marking are performed in the preoperative holding area, whereas the "time out" is performed in the operating room (OR) as a final recapitulation immediately prior to surgery [4-6]. All three steps of the Universal Protocol are designed to ensure correct patient identity, correct procedure, and correct surgical site. In addition, the time out was recently expanded to include the verification of correct patient positioning, availability of relevant documents, diagnostic images, instruments and implants, and the need for preoperative antibiotics and other essential medications, e.g. the use of beta-blockers [7]. Of note, this protocol also applies to clinical settings outside the operating room, for any invasive procedure which requires a patient's consent.

## Wrong site surgery – the "horror" is far from over

Wrong site and wrong patient procedures have been defined as "never-events" which are theoretically 100% preventable and thus should never occur (table 1). Despite the widespread implementation of the Universal Protocol since 2004, multiple reports have documented continued occurrence of wrong site and wrong patient procedures in the United States [8-18]. Clarke *et al. *published an analysis of hospital reports on reported wrong site, wrong patient, and wrong procedure surgery in the state of Pennsylvania during a 30-month period from 2004–2006 [10]. The authors detected 427 reports of wrong-site occurrences, of which 56% were "near miss" events. In their series, a formal "time out" was unsuccessful in preventing wrong-site surgery in 31 cases [10]. Jhawar and colleagues performed a national survey to estimate the incidence of wrong side and wrong level craniocerebral and spinal surgery among practicing neurosurgeons in the Unites States [9]. Among the 138 responding neurosurgeons, 25% admitted to having performed incisions on the wrong side of the head at one point during their careers. In addition, 35% of all neurosurgeons who had been in practice for more than 5 years disclosed a wrong level lumbar spine procedure at some point of their careers [9]. Seiden and Barach reviewed the National Practitioner Data Bank and additional closed claims databases for wrong site procedures [8]. The authors concluded that wrong site surgery continues to occur approximately 1,300 to 2,700 times annually in the United States [8]. In contrast to this reported high frequency, Kwaan *et al. *proposed that wrong site procedures are infrequent, and may not be preventable by current site-verification protocols [19]. The authors reviewed all wrong site surgery cases (except for wrong level spine surgeries) reported to a malpractice insurer between 1985 and 2004. A total of 25 wrong site procedures were detected during a 20-year study period on 2,826,367 operations. These data imply an exceptionally rare incidence of wrong site surgery of 1 case in 112,994 operations [19]. This study was, however, severely criticized for flaws in design and interpretation, most importantly based on selection bias due to the exclusive restriction of wrong site cases to malpractice claims, since many wrong site procedures never turn into a claim [16].

**Table 1:** Serious reportable surgical events ("never-events"), as defined by the National Quality Forum (NQF consensus report, update 2006)

**Surgical "never-events"**
1. Surgery performed on the wrong body part.

2. Surgery performed on the wrong patient.

3. Wrong surgical procedure performed on a patient.

4. Unintended retention of a foreign object in a patient after surgery or other procedure.

5. Intraoperative or immediate postoperative death in an ASA class I patient.

Independent of the ongoing debate related to the true incidence of wrong patient and wrong site surgery, only a culture of "zero tolerance" will keep patients maximally safe.

## Limitations of the Universal Protocol

As outlined above, the Universal Protocol has not yet proven to be 100% protective against the occurrence of wrong site and wrong patient surgery since its mandatory implementation 5 years ago. Pitfalls and limitations which may fail the system are hidden in each component of the protocol. Most importantly, the degradation of the Universal Protocol to a robotic-hackneyed type ritual will distract from the requisite focus. Inadequate or inaccurate site marking represents another major pitfall leading to wrong site surgery. The "time out" *per se *should never absolve the lead surgeon from taking full responsibility in ensuring by all available means that the correct procedure is performed at the correct site in the correct patient. The continuing expansion of the "time out" to include secondary safety issues, such as antibiotic and venous thromboembolism prophylaxis (as implemented in the so-called "expanded surgical time-out"), further dilutes the essence and distract from the protocol's core mission [7,20]. Another inherent risk factor for wrong site surgery is represented by the situation of multiple simultaneous procedures performed on the same patient, thus obscuring the focus of the "time out" for one defined procedure. In addition, specific anatomic locations may represent "black boxes" for adequate site marking, thus increasing the risk of wrong site procedures. Finally, a significant loophole in the system is the lack of a "universal" implementation of the Universal Protocol. This notion is supported by recent, unpublished data which revealed that non-surgical specialties, e.g. internal medicine and family practitioners, are predominantly involved in the etiology of wrong patient surgery and contribute significantly to patient harm after wrong site procedures (Stahel PF, Mehler PS, et al., *unpublished data*). Based on these findings, a strict adherence to the Universal Protocol must be advocated to be expanded also to non-procedural specialties in the future.

## The pre-procedure verification process

About 20%–30% of all wrong site and wrong patient procedures have their genesis before patient admission to the hospital. Potential scenarios include inaccurate clinic note dictations related to a wrong side, the mislabelling of radiographs or other diagnostic tests, or a mix-up of patients with similar or identical names. The rationale for conducting a pre-procedure verification process is to confirm: (1) patient identity, (2) the nature of the planned procedure, and (3) the exact surgical site. Each patient is unequivocally identified by an identification bracelet which includes the patient's name, birth date, and a medical record number. The surgical consent form is then presented to the patient, with the intended surgical procedure and the name of the responsible surgeon being spelled out. The patient signs the consent form only with all information proven correct. Surgical site marking is performed as part of the pre-procedure verification process, by the surgeon, in the preoperative holding area. The pre-verification process further ensures presence and adequacy of all relevant documents, including a current history and physical exam, and a written informed consent. Finally, the team's understanding of the planned procedure is confirmed to be consistent with the patient's expectations. A checklist is used to review and verify that all documents and pertinent information are available, accurate, and completed, prior to moving the patient to the operating room.

## Surgical site marking – pitfalls

**Figure 1:**
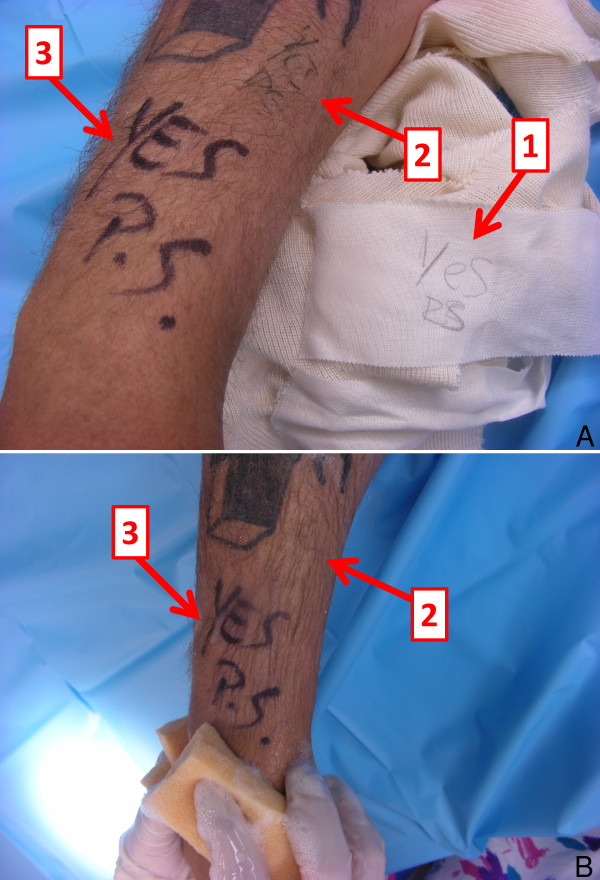
**Clinical example of correct versus incorrect modalities of surgical site marking**. **A**: This patient was scheduled for a surgical procedure on his right forearm. The intern marked and initialed the site on the dressing, which came off prior to surgery (1). The resident corrected the mistake by marking the surgical site on skin, using a regular pen (2). Neither the marking, nor the initials, are well legible (2). Finally, the site was again marked and initialed by the attending surgeon with a permanent marker (3). **B**: During the surgical preparation, the site marking with the regular pen was washed off immediately (2), whereas the permanent marker remained visible throughout the surgical preparation (3). This example emphasizes the crucial importance of using a permanent marker, large and well legible letters, and to sign the marking with the surgeon's initials. "YES" is the designated, standardized identifier for the correct surgical site at Denver Health Medical Center.

Inadequate or inaccurate surgical site marking – including the erroneous marking of the wrong side/site, imprecise marking of the correct site, and inadequate modality of site marking – represent a major risk factor for wrong site surgery (Figure 1).

Examples of such adverse circumstances include:

- The relegation of site marking and time out to a junior member of the surgical team, e.g. to an intern, or to a physician who will not be personally present during the operative procedure.

- Wrong modality of marking the correct side, e.g. using an "X" which may be misunderstood as "not this side".

- Marking of the wrong side/site based on misleading pre-procedure documentation, e.g. erroneous clinic note dictation, faulty documentation in chart and consent form, and mislabelling of diagnostic studies, e.g. X-rays.

- Imprecise site marking. Case examples include: (1) Marking the correct joint without specifying the operative site, leading to wrong-site collateral ligament release (medial vs lateral); (2) Marking the correct hand, without specifying the correct finger and joint, leading to wrong-level joint fusion (DIP vs PIP); (3) Marking the correct spinal level on skin, but fusing the wrong level after surgical dissection down to the spine.

- The use of non-permanent markers will increase the risk of wrong site surgery, since surgeons may operate on non-marked sites under the faulty assumption that the site marking had been wash off during the surgical preparation.

- Additional marking of the contralateral side (e.g. "no" or "not this side") is considered obsolete, since this will create confusion and increase the risk of wrong-sided surgery.

- Residual marks from a previous surgery in the same patient may be misleading and distract from the correct surgical site for an additional intervention (e.g. polytrauma patient with multiple fractures stabilized at different time-points).

- Inability (or contraindication) to mark the surgical site.

Moreover, specific instances may not allow surgical site marking, for technical or anatomic reasons. For example, site marking is impracticable on mucosal surfaces and on the teeth. Site marking is furthermore considered contraindicated in premature infants, due to the risk of inducing a permanent tattoo on the skin. Some surgical sites are inaccessible to accurate external marking. Exemplary circumstances include visceral surgery (internal organs), neurosurgery (brain, spine), interventional radiology (vascular procedures), and orthopaedic surgery on the torso (pelvis, spine). Rarely, patients may refuse surgical site marking for cosmetic or other personal reasons.

A defined, alternative process must be in place for all above-mentioned circumstances. Radiological diagnostics may need to be consulted pre- and intraoperatively to determine the surgical site with accuracy. For example, spine surgeons must ensure the correct intervertebral level with a needle using intraoperative fluoroscopy in order to avoid a wrong-level spine fusion. Similarly, general surgeons may have to rely on a preoperative or on-table cholangiogram to ensure clipping the correct bile duct, i.e., the cystic duct instead of common bile duct. Furthermore, interventional radiology procedures pose a similar risk for wrong site surgery, e.g. by the erroneous coiling of a wrong artery. Finally, neurosurgical interventions on the wrong part of the brain keep being reported in regular intervals [9,14,21]. Unlike symmetric external body parts, such as extremities, eyes and ears, these "hidden" surgical sites may not be easily identified, confirmed and marked prior to surgery. Thus, these particular circumstances may mandate an accurate intraoperative localization under fluoroscopy, in conjunction with a careful evaluation of the surgical site by additional preoperative diagnostics, such as CT, MR, angiography, or cholangiography.

While it is currently not mandatory to mark the surgical site in 100% of patients, efforts should be made to mark all surgical sites whenever possible. This includes marking the abdominal wall or the chest for intended procedures on internal organs, which is aimed at increasing the surgeon's awareness and focus on performing correct site surgery.

## Surgical site marking – the correct way

Based on the available recommendations and standards, and on lessons learned from failures and complications, we recommend taking the following parameters into account to increase the accuracy and safety of surgical site marking:

- Site marking must be performed by a licensed practitioner who is a member of the surgical team and will be present during the surgical "time out" and during the procedure. Under ideal circumstances, site marking should be performed by the lead surgeon.

- Mark the site in the preoperative holding area, before moving the patient to the operating room or to any other location where a procedure will be performed.

- Involve the patient in the site marking process whenever possible.

- Site marking must be unambiguous, using clearly defined terminology such as "YES", "GO", "CORRECT", or "CORRECT SITE". The exact marking modality must be defined and consistent within a specific institution.

- Responsibility of site marking should be confirmed by adding the surgeon's initials. An exception is a surgeon's name with the initials "N.O." since this abbreviation may be confounded with "no" and imply that the marked site should not be operated on.

- Site marking must be applied by indelible ink on skin, using permanent markers. The use of temporary or removable markers, e.g. using stickers or marking on casts or dressings, is not feasible.

- Site marking must be resistant to the surgical preparation process and remain visible at the time of skin incision.

- Sterility of the marking ink or marking pen is not required; the published literature has shown that the use of non-sterile markers does not increase the risk of postoperative infections [22-25].

- Apply the marking at or near the incision site. The side, level, and location of the procedure must be unequivocally defined by the marking, whenever possible. Marking takes into consideration the side (laterality), surface (flexor/extensor, medial/lateral), the spinal level, and the specific digit or lesion to be operated on.

- Increased awareness in all cases where precise site marking is not possible (see above).

- Knowledge of contraindications for surgical site marking, including premature infants (risk of permanent tattoo), mucosal surfaces, teeth, and patients refusing a surgical site marking for personal reasons.

- Implementation of defined alternative processes for any circumstance where surgical site marking is not feasible. Include pre- and intraoperative radiological diagnostics to increase the accuracy of determining the correct surgical site (e.g. spinal level marking with a needle, intraoperative arteriogram or cholangiogram, etc.)

## The surgical "time out"

The surgical "time out" represents the last part of the Universal Protocol and is performed in the operating room, immediately before the planned procedure is initiated. The "time out" represents the final recapitulation and reassurance of accurate patient identity, surgical site, and planned procedure. In addition, the correct patient positioning, the need for perioperative antibiotics, presence of allergies, and the availability of relevant documents and diagnostic tests, instruments, implants and other pertinent equipment are confirmed during this time. The following parameters are key to success for a surgical "time out":

- A "time out" is called by any member of the surgical team, but usually by a specifically designated person, e.g. the circulation nurse.

- Ideally, the patient should be awake and participate in the verification process of patient identity, surgical site, and planned procedure (so-called "awake time out").

- The "time out" process must be standardized at every institution.

- The immediate members of the procedure team (i.e. surgeon, anaesthesia provider, circulating nurse, and operating room technician) must actively participate in the "time out".

- During the "time out", all other activities are suspended to an extent which does not compromise patient safety.

- The "time out" must be repeated intraoperatively for every additional procedure performed on the same patient.

## Conclusion

The Universal Protocol was mandated by the *Joint Commission *5 years ago with the aim of increasing patient safety by avoiding procedures at the wrong site or in the wrong patient. Despite widespread implementation, this standardized protocol has failed to prevent such severe "never-events" from occurring. Potential technical pitfalls and loopholes in the system are addressed in this editorial. All healthcare institutions (not just in the United States) across all specialties (not just surgical disciplines) should commit to adherence to the Universal Protocol as a standardized quality assurance tool. Current limitations, such as tolerated differences in site marking modalities across institutions, should be addressed by a more "universal" standardization of the process. The ultimate determinant of the success of this system is the entire team's commitment to make it work, and to abort the process to start over if any objections or inconsistencies are encountered. Patients must be involved in the site marking process and educated to inquire of their surgeons whether a formal "time out" procedure will occur in the surgical suite. Our long-term aim must be directed towards educating ourselves, the next generation of health care providers, and our patients, to strive for an enduring and unfailing patient safety culture. The onus is on us.

## Competing interests

The authors declare that they have no competing interests.

## Authors' contributions

All authors contributed equally in the design and writing of this editorial.
